# 24h Urinary Protein Levels and Urine Protein/Creatinine Ratios Could Probably Forecast the Pathological Classification of HSPN

**DOI:** 10.1371/journal.pone.0127767

**Published:** 2015-05-21

**Authors:** Qing Ye, Shi-qiang Shang, Ai-min Liu, Ting Zhang, Hong-qiang Shen, Xue-jun Chen, Jian-hua Mao

**Affiliations:** 1 The Children’s Hospital of Zhejiang University School of Medicine, Hangzhou, PR China; 2 Zhejiang Key Laboratory for Diagnosis and Treatment of Neonatal Diseases, Hangzhou, PR China; 3 Zhejiang Chinese Medical University, Hangzhou, PR China; University of São Paulo School of Medicine, BRAZIL

## Abstract

This study aimed to assess the relevance of laboratory tests in Henoch-Schönlein purpura nephritis (HSPN) classification, and determine accurate classification factors. This prospective study included 694 HSPN patients who underwent ultrasound-guided percutaneous renal biopsy (PRB). Renal specimens were scored according to International Study of Kidney Disease in Children (ISKDC) classification. Meanwhile, blood samples were immediately collected for laboratory examination. The associations between laboratory parameters and HSPN classification were assessed. Significant differences in levels of serum Th1/Th2 cytokines, immunoglobulins, T-lymphocyte subsets, complement, and coagulation markers were obtained between HSPN patients and healthy children. Interestingly, 24h urinary protein (24h-UPRO) levels and urine protein/urine creatinine ratios could determine HPSN grade IIb, IIIa, and IIIb incidences, with areas under ROC curve of 0.767 and 0.731, respectively. At 24h-UPRO >580.35mg/L, prediction sensitivity and specificity were 75.2% and 70.0%, respectively. These values became 53.0% and 82.3%, respectively, with 24h-UPRO exceeding 1006.25mg/L. At urine protein/urine creatinine > 0.97, prediction sensitivity and specificity were 65.5% and 67.2%, respectively, values that became 57.4% and 80.0%, respectively, at ratios exceeding 1.2. Cell and humoral immunity, coagulation and fibrinolytic systems are all involved in the pathogenesis of HSPN, and type I hypersensitivity may be the disease trigger of HSPN. 24h-UPRO levels and urine protein/creatinine ratios could probably forecast the pathological classification of HSPN.

## Introduction

Henoch-Schönlein Purpura (HSP) is a small vessel vasculitis with variable clinical features such as skin purpura, arthritis and/or arthralgia, kidney damage, and gastrointestinal disease. Renal HSP, known as Henoch-Schönlein purpura nephritis (HSPN), is the most serious complication, and a key factor affecting patient prognosis [1, 2]. The extent of renal injury is important in HSPN prognostic evaluation and early individualized therapy. However, clinical manifestations do not always correlate with the severity of renal pathological findings in HSPN children [3]. Thus, classifying renal pathology by kidney biopsy is the gold standard to evaluate renal involvement. However, renal biopsy is an invasive operation, not accepted by all patients; in addition, the pathological type changes as the disease progresses. Therefore, using non-invasive methods to predict HSPN type is of great value. Here, we analyzed the relevance between laboratory parameters and HSPN classification, to identify favorable predictors.

## Materials and Methods

### Study Subjects

This was a prospective observational study carried out from February 1992 to December 2014. It was approved by the ethics committee of The Children Hospital of Zhejiang University School of Medicine. Parents or guardians signed written informed consent forms for all minors involved in this study. Children meeting the following criteria were included: (1) age < 18 years; (2) diagnosis of HSPN by both two doctors according to KDIGO criteria [4]. Patients with any other pre-existing disease were excluded from the study. Four hundred healthy children were randomly selected as normal controls. In the acute phase of HSPN, blood samples were collected for serum Th1/Th2 cytokine, complement, and immunoglobulin levels, T lymphocyte subset assessment, blood routine test, and coagulation spectrum and CRP level determination. Meanwhile, urine samples were obtained for creatinine and protein quantitation, and white blood cell and red blood cell counts.

Proteinuria was defined as urinary protein excretion greater than 150 mg/24h; haematuria was considered for more than 5 red blood cells per high magnification field under the microscope after centrifugation; leucocyturia was considered for more than 5 white blood cells per high magnification field under the microscope after centrifugation.

### Serum cytokine levels and T-cell profiling

These parameters were determined as previously described [5, 6]. Briefly, blood samples were centrifuged at 1,000g for 20 min at 20°C after clotting to prepare serum. Then, serum Th1 and Th2 cytokine levels were assessed by 320 flow cytometry immediately. The concentrations of IL-2, IL-4, IL-6, IL-10, tumor necrosis factor (TNF)-a, and interferon (IFN)-γ were assessed using the CBA Human Th1/Th2 Cytokine Kit II (BD Biosciences, San Jose, CA).

T-cell subsets were detected by multicolor flow cytometry (FACSCalibur, BD, USA) using blood samples containing heparin. Mouse anti-human CD3-FITC, CD4-APC, and CD8-PEmonoclonal antibodies, and other reagents were purchased from BD. Data was analyzed by the MultiTEST software.

### Assessment of immunoglobulin, complement and CRP levels

Immunoglobulin and complement levels were evaluated on a SIEMENSBN-II specific protein analyzer (SIEMENS, Germany). CRP levels were measured on a QuikRead go (Orion Diagnostica, Finland) with QuikRead go CRP kits.

### Detection of urine protein and creatinine levels, and white blood cell and red blood cell counts

Urinary protein and creatinine amounts were measured on a Roche Modular P800 biochemical analyzer. Blood cell numbers in urine were determined using a SYSMEX UF-1000 automatic urinary sediment analyzer.

### Blood routine test and coagulation spectrum determination

Blood routine test was carried out on a SYSMEX automated hematology analyzer (XS 800i). Coagulation spectrum was obtained with a SYSMEX CA-1500.

### Renal biopsy grading

All patients were operated by ultrasound-guided percutaneous renal biopsy (PRB), and samples histopathologically graded as types I to VI according to the ISKDC classification standard by a professional pathologist: Type I, minimal glomerular abnormalities; Type II, mesangial proliferation without crescents; Type III, focal segmental (IIIa) or diffuse (IIIb) mesangial proliferation with<50% crescents; Type IV, mesangial proliferation with 50–70% crescents; Type V, mesangial proliferation with>75% crescents; Type VI, membrano-proliferative-like lesions.

### Statistical analysis

Comparisons between two groups were performed using χ^2^ or Fisher’s exact tests for categorical variables, and Mann–Whitney U test for continuous variables. Pearson correlation was used to assess the association between two variables. All statistical analyses were performed with the SPSS 18.0 software. *P*<0.05 was considered statistically significant.

## Results

### Patient characteristics

This prospective study included 694 patients with HSPN who were admitted at the Nephrology Department, Children’s Hospital of Zhejiang University School of Medicine, Hangzhou, china, from February 1992 to December 2014. Patients averaged 8.96 (ranging from 3 to 17) years old. There were 332 boys and 362 girls, suggesting that both genders are equally affected by HSPN (P>0.05). Additionally, no statistical differences in gender and age (P>0.05) were obtained between the HSPN and control groups.

According to ISKDC recommended diagnosis criteria, there were 54 type I (7.8%), 122 type IIa (17.6%), 215 type IIb (31.0%), 102 type IIIa (14.7%), 191 type IIIb (27.5%), 7 type IV (1.0%), and 3 type V (0.4%) cases. No significant differences in ages were obtained among different HSPN pathological types (χ2 = 3.03, P = 0.553, Table 1).

**Table 1:** The levels of laboratory indexes from HSPN group and normal control group.

Parameters	normal control group	Ⅰ	Ⅱa	Ⅱb	Ⅲa	Ⅲb	*P* value
case	400	54 (7.8%)	122 (17.6%)	215 (31.0%)	102 (14.7%)	191 (27.5%)	
girls/boys	212/188	33/21	73/49	95/120	53/49	103/88	0.553
age (year)	8.75(4.1→17.0)	7.0(5.0→11.0)	8.0(5.0→14.0)	9.0(3.0→17.0)	8.0(4.0→14.0)	9.0(4.0→16.0)	0.621
eGFR (CKD-EPI) (ml/min/1.73m^2^)	176.1(146.8→229.8)	166.3(135.9→189.7)	163.2(137.2→199.2)	167.5(138.6→242.7)	158.0(142.2→181.7)	158.4(138.7→190.4)	<0.01
urine creatinine (umol/L)	7774.5(3225.0→18229.0)	6363.0(1937.4→20275.0)	7185.9(109.2→32525.0)	7420.9(1442.3→24338.0)	8134.5(569.0→20985.0)	8321.6(1392.0→237777.0)	0.732
urine protein/urine creatinine	0.1(0.01→0.2)	0.7(0.1→9.7)	0.5(0.1→4.8)	1.3(0.1→7.1)	1.4(0.1→4.1)	1.6(0.1→6.8)	<0.01
24 hour urinary protein quantity (mg/L)	32.6(2.0→567.0)	263.4(47.0→4444.0)	311.4(12.0→4513.0)	888.4(89.0→5422.0)	1014.7(11.0→4523.0)	1171.6(188.0→8025.0)	<0.01
IL-2 (pg/mL)	5.9(2.7→7.8)	2.5(1.0→4.0)	2.2(1.1→14.8)	2.1(1.0→6.4)	2.3(1.0→6.7)	2.4(1.0→6.0)	<0.01
IL-4 (pg/mL)	2.7(1.1→4.0)	1.7(0.9→4.8)	1.7(0.6→5.2)	2.0(0.6→7.2)	2.1(0.4→5.9)	2.3(0.8→4.8)	<0.01
IL-6 (pg/mL)	4.2(1.2→8.5)	5.6(1.2→178.2)	4.3(1.1→2247.8)	3.1(1.0→135.6)	3.4(1.5→239.8)	3.3(1.6→147.9)	0.181
IL-10 (pg/mL)	2.4(1.3→9.9)	3.5(1.6→12.3)	3.7(1.6→10.0)	3.0(1.1→31.0)	3.2(1.0→18.1)	3.0(1.1→21.9)	<0.01
TNF-α (pg/mL)	2.3(1.0→3.1)	2.7(1.5→46.9)	2.5(1.0→235.7)	2.3(1.0→46.7)	2.6(1.0→44.2)	2.5(1.0→70.4)	0.015
INF-γ (pg/mL)	4.4(3.0→8.0)	4.4(1.0→10.0)	3.5(2.0→52.0)	4.0(1.0→19.0)	3.8(2.0→12.0)	4.2(1.0→41.0)	0.022
CD3+ (% of positivity)	64.4(56.6→69.7)	65.0(48.6→78.1)	65.8(43.1→82.5)	67.1(47.8→82.3)	63.4(41.8→81.2)	66.3(40.1→80.8)	0.338
CD4+ (% of positivity)	34.8(28.5→46.4)	36.5(26.1→42.5)	31.8(16.9→51.7)	33.4(9.9→48.3)	30.0(15.5→44.3)	30.1(17.0→45.1)	<0.01
CD8+ (% of positivity)	22.5(16.6→29.8)	24.7(17.0→34.9)	27.5(13.2→38.1)	25.4(15.4→45.77)	25.6(16.1→43.0)	27.1(15.0→46.5)	<0.01
CD4+/CD8+	1.6(1.0→2.8)	1.5(1.1→2.2)	1.2(0.5→2.3)	1.2(0.3→2.2)	1.0(0.5→2.4)	1.1(0.4→2.1)	<0.01
IgE (IU/mL)	28.2(4.0→98.0)	81.7(5.0→450.0)	43.9(2→300.0)	55.6(4.0→2430.0)	55.5(4.0→1870.0)	50.7(7.0→687.0)	<0.01
IgG (g/L)	9.6(3.2→17.6)	9.3(6.3→15.2)	9.4(1.3→17.4)	8.6(2.1→17.3)	9.3(2.9→16.7)	8.6(1.1→22.6)	0.34
IgA (g/L)	0.9(0.02→3.8)	1.5(0.5→2.6)	2.1(0.8→3.5)	1.9(0.3→3.8)	2.0(0.9→4.4)	2.1(0.6→5.1)	<0.01
IgM (g/L)	0.8(0.4→2.0)	1.2(0.9→1.8)	1.3(0.3→2.8)	1.1(0.2→2.8)	1.2(0.2→3.5)	1.2(0.6→2.8)	<0.01
C3 (g/L)	1.1(0.7→1.4)	1.0(0.2→1.4)	1.2(0.7→1.6)	1.2(0.9→1.6)	1.2(0.6→1.9)	1.2(0.7→3.0)	0.049
C4 (g/L)	0.2(0.1→0.4)	0.3(0.2→0.4)	0.3(0.1→0.5)	0.3(0.1→0.6)	0.3(0.1→1.0)	0.3(0.1→0.6)	0.006
D-dimer (mg/L)	20.5(5.0→51.0)	83(48.0→5860.0)	99.5(47.0→591.0)	110.0(49→12200)	151.0(30.0→3860.0)	183.5(48.0→10710.0)	0.015
FIB (g/L)	2.2(2.0→4.0)	3.0(2.0→4.0)	2.7(1.0→4.0)	2.7(2.0→5.0)	2.7(2.0→5.0)	2.8(1.0→5.0)	<0.01
PLT (×10^9^/L)	252.0(116.0→393.0)	330.0(180.0→441.0)	338.5(109.0→464.0)	321.0(165→741)	362.5(3.0→628.0)	304.5(176.0→700.0)	<0.01
PDW	15.9(10.3→17.2)	16.7(16.1→19.4)	16.6(9.9→21.8)	16.0(8.8→19.0)	16.3(8.5→18.9)	16.2(8.7→20.3)	<0.01
CRP (mg/L)	3.0(1.0→7.0)	1.0(1.0→48.0)	1.0(1.0→15.0)	1.0(1.0→33.0)	1.0(1.0→13.0)	1.0(1.0→8.0)	<0.01
Urine RBC (/uL)	3.0(0.0→22.0)	100.0(5.0→1000.0)	200.0(16.0→1384.0)	118.9(0.0→1538.0)	186.5(8.0→1000.0)	167.6(22.0→2473.0)	<0.01
Urine WBC (/uL)	5.1(0.0→10.3)	5.0(0.0→20.5)	5.1(0.0→153.8)	3.0(0.0→66.7)	7.0(0.0→92.3)	10.3(0.0→153.8)	0.003

*P* value: All types of purpura nephritis patients as a group compared with normal controls. Range and median values are represented for each group.

### Laboratory parameters in HSPN children with different pathological types

Compared with healthy children, HSPN patients had significantly lower levels of CD4+ (Z = -4.135, P<0.01) and higher CD8+ amounts (Z = -4.931, P <0.01), and therefore a significant reduction in the CD4+/CD8+ ratio (Z = -5.975, P <0.01). There was no difference in CD3+ cell amounts (Z = -0.959, P = 0.338). However, none of these parameters (CD3+, CD4+, and CD8+ levels and CD4+/CD8+ ratios) showed a significant difference among various HSPN pathological groups. Interleukin-2 (IL-2) (Z = -10.087, P<0.01), IL-4 (Z = -4.704, P<0.01) and Interferon-γ (INF-γ) (Z = -2.295, P = 0.022) levels were significantly decreased in HSPN patients compared with healthy children, while IL-10 (Z = -3.545, P<0.01) and Tumor Necrosis Factor-α (TNF-α) (Z = -2.424, P = 0.015) amounts were significantly increased. There was no significant difference in IL-6 levels (Z = -1.339, P = 0.181). However, no significant differences were obtained in serum cytokines levels among various HSPN pathological groups. Serum IgE (Z = -4.694, P<0.01), IgA (Z = -8.655, P<0.01) and IgM (Z = -4.667, P<0.01) levels in the HSPN group were significantly higher than in healthy children (P<0.05), while no difference in IgG levels was observed (Z = -0.954, P = 0.340). What is more, type I HSPN patients had the highest serum IgE levels (Z = -2.254, P = 0.024) and relatively lower IgA amounts (Z = -2.252, P = 0.024) compared with other HSPN types. Serum complement C3 (Z = -1.966, P = 0.049) and C4 (Z = -2.744, P = 0.006) levels were significantly higher in HSPN patients than in healthy children. Type I HSPN patients showed lowest C3 levels (Z = -2.130, P = 0.033); no significant difference in C4 levels among HSPN types was obtained (Z = -0.481, P = 0.630). Significantly higher levels of PLT (Z = -5.185, P<0.01), FIB (Z = -4.835, P<0.01) and D-dimer (Z = -2.437, P = 0.015) were observed in HSPN patients compared with healthy children, while there was no difference among various HSPN pathological groups (Table 1).

### Correlation between immune indexes and leucocyturia, hematuria and proteinuria

Correlation analyses showed that red blood cell (RBC) amounts in urine were negatively correlated with IL-2 levels in children with HSPN having hematuresis (r = -0.255, P<0.01) and positively correlated with PLT (r = 0.147, P = 0.017) and complement C4 (r = 0.176, P = 0.004) levels. White blood cell (WBC) numbers were positively correlated with complement C3 levels (r = 0.255, P<0.01). Interestingly, urinary protein contents in children with HSPN were negatively correlated with IL-2 (r = -0.320, P<0.01), CD3+ cell (r = -0.163, P = 0.008), CD4+ cell (r = -0.209, P = 0.001), CD4+/ CD8+ cell (r = -0.180, P = 0.003) and IgG (r = -0.191, P = 0.002) amounts, and positively correlated with PLT (r = 0.132, P = 0.033) and IgA (r = 0.192, P = 0.002) levels.

### Prediction of HSPN pathological classification

Compared with healthy children, 24h urinary protein levels were significantly higher (Z = -10.543, P<0.01) in HSPN patients; however, no significant differences in urine protein contents (Z = -0.160, P = 0.873) and urine protein/creatinine ratios (Z = -0.780, P = 0.435) were obtained between HSPN types I and IIa. In addition, no significant differences in urinary protein contents(χ^2^ = 3.327, P = 0.190)and urine protein/creatinine ratios(χ^2^ = 2.911, P = 0.233)were obtained among HSPN types IIb, IIIa and IIIb. However, significant differences in 24h urinary protein contents (Z = -6.102, P<0.01) and urine protein/creatinine ratios (Z = -5.248, P<0.01) were obtained between the two groups of HSPN patients (types I and IIa vs. types IIb, IIIa, and IIIb): both 24h urinary protein contents and urine protein/creatinine levels were higher in HSPN types IIb, IIIa, and IIIb compared with types I and IIa patients.

**Fig 1:**
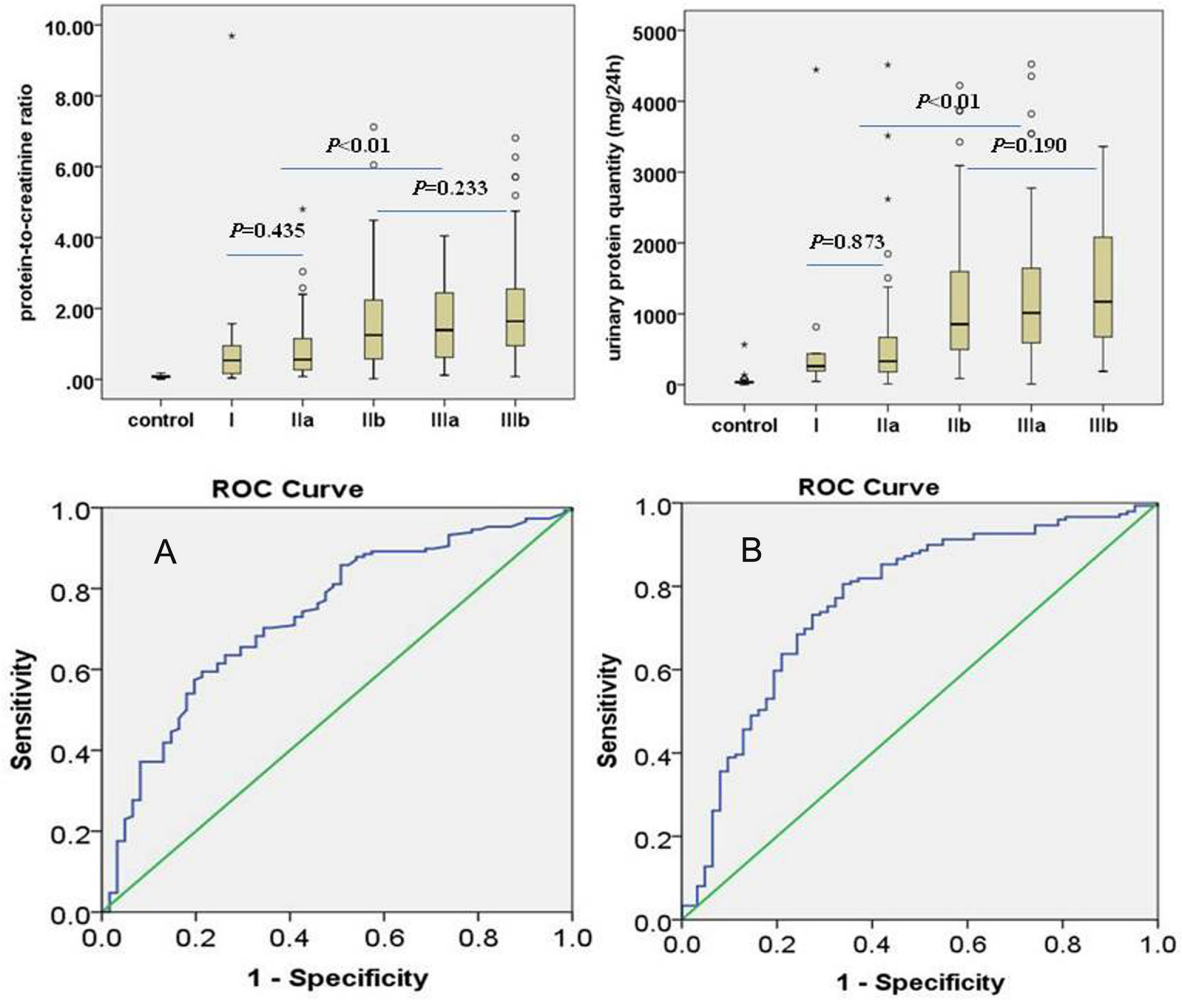
Ability of laboratory parameters to predict pathological types in children with Henoch-Schönlein purpura nephritis. A: ROC curve for predictive value of 24h urinary protein (24h-UPRO) amounts for HPSN typed IIb, IIIa, and IIIb. B: ROC curve for predictive value of urine protein/ creatinine ratios for HPSN typed IIb, IIIa, and IIIb.

24h urinary protein (24h-UPRO) amounts and urine protein/ creatinine ratios were evaluated for their predictive value of HPSN typed IIb, IIIa, and IIIb incidence; areas under ROC curve were 0.767 and 0.731, respectively. At 24h-UPRO >580.35 mg/L, prediction sensitivity and specificity were 75.2% and 70.0%, respectively. These values became 53.0% and 82.3%, respectively, with 24h-UPRO exceeding 1006.25 mg/L (Fig 1). At urine protein/urine creatinine > 0.97, prediction sensitivity and specificity were 65.5% and 67.2%, respectively, values that became 57.4% and 80.0%, respectively, at ratios exceeding 1.2 (Fig 1).

## Discussion

In this study, CD4+ levels in children with HSPN were decreased, while CD8+ amounts were increased, leading to a remarkable decline in CD4+/CD8+ ratios. In addition, urine protein contents in children with HSPN were negatively correlated with CD4+ cell amounts and CD4+/CD8+ ratios, suggesting a T cell dysfunction and a hyperfunctional cellular immunity in HSPN patients.

T helper (Th) cells play an important role in the immune system *in vivo*. They are composed of several cell subsets. Among them, Th1 cells are responsible for cell-mediated immune responses, while Th2 are associated with humoral-mediated immunity. Th1 cells secrete Th1 cytokines such as IFN-γ and TNF-α; Th2 cells produce IL-4, IL-6 and IL-10 that are known as Th2 cytokines. Cytokine secretion may contribute to the cross-regulation or mutual inhibition of these subsets. In the initial phase of the immune response, if a certain subset is selected, such Th cells will enhance their advantages as positive feedback, while suppressing the development of the other subgroup. Thl/Th2 imbalance is believed the main cause of certain disease occurrence or progression [7]. In the acute phase of HSPN, a Thl/Th2 imbalance is found in blood: the Th1 cytokines IL-2 and IFN-γ are reduced, while IL-4, a Th2 cytokine, is even more pronouncedly decreased, which presents an advantage for the Th1 subset. Tsuruga et al. also showed that HSPN patients with proteinuria have increased T-bet and reduced GATA-3 expression levels in the urinary sediment, indicating a possible shift in Th1/Th2 balance towards Th1 predominance [8]. This further shows that cellular immunity plays an important role in the pathogenesis of HSPN.

IL-2 levels in patients were reduced, while serum levels of immunoglobulins like IgE, IgA and IgM were remarkably increased; however, IgG amounts were not significantly changed. Likely, the lack of IL-2 resulted in non-effective inhibition of B cell maturation and non-effective natural killer (NK) cell activation, which causes an abnormal humoral immune response and reduced foreign antigen scavenging activity. Consequently, there is an increased secretion of immunoglobulins that are deposited in the kidney, causing an immune complex-mediated injury and immune-based immune complex vasculitis, which finally leads to renal injury.

Urinary protein contents in children with HSPN were positively correlated with IgA levels. HSPN type I patients had the highest IgE levels. This suggests that IgE-mediated type I hypersensitivity may play an important role at the beginning of HSPN, and may be the disease trigger. This finding also supports the hypothesis that allergic purpura originates from allergy. Our previous findings [9] also showed significantly higher serum IgE levels in patients with acute phase HSP compared with healthy children, in agreement with the findings [10] that IgE-sensitized mast cells promote the release of vasoactive substances, and increase vascular permeability in favor of IgA circulating immune complex (CIC) deposition [11]. IgA levels in HSPN type I patients were significantly higher than in healthy children, and maintained at high levels when the disease progresses to type IIa or further stages. *In vitro* studies have shown that polymeric IgA forms (pIgA) bind to mesangial cells to enhance cell proliferation, cytokine release, and extracellular matrix (ECM) production [12, 13]. Thus, IgA is one of the most important risk factors in renal lesions development and progression in HSP. In addition, serum IgM levels were significantly increased in both types I and IIa HSPN patients then decreased significantly, indicating that infection is involved in the early stage of HSPN. Meanwhile, the slight changes observed in IL-6 and CRP levels exclude bacterial infection, thereby indicating a high probability of viral infection.

Serum complement C3 and C4 levels were significantly increased, suggesting the activation of the complement system. Activation of the complement cascade is triggered by one of the following pathways: classical, alternative and mannose-binding lectin (MBL) pathways. All three pathways converge to cleave component C3, which subsequently initiates activation of the terminal complement pathway, and formation of a series of complement protein fragments with important biological effects as well as the membrane attack complex (MAC). MAC is a product of complement activation with strong pathogenicity. It can lyse cells, stimulate cytokine production and injure glomerular epithelial, mesangial, and endothelial cells, leading to a direct or indirect glomerular cell damage. In HSPN, the tissue immuno-pathological damage caused by the activated complement is mainly through the alternative and mannose-binding lectin (MBL) pathways [14]. It is known that C3 deposition is related to the severity of proteinuria in HSPN children [15]. Although hypocomplementemia has been reported in some patients with HSPN [16], our findings indicate that except for mild cases (type I patients) with no significant increase of complement C3 and C4, complement levels were significantly higher when the disease progresses to type IIa or further stages. In addition, correlation analyses showed that red blood cell (RBC) amounts in urine are positively correlated with complement C4 levels; meanwhile, white blood cell (WBC) numbers in urine were positively correlated with complement C3 levels, suggesting that the complement system participates in the renal injury of HSPN.

Serum PLT, FIB and D-dimer levels are increased significantly in HSPN. Correlation analyses showed that urine red blood cell amounts and protein contents are positively correlated with PLT levels. Multiple clinical and experimental studies have shown that fibrinogen deposition is involves in crescent formation; therefore, immunosuppressants combined with anticoagulants have been suggested in the treatment of such diseases, including warfarin, dipyridamol and acetylsalicylic acid [17]. Thus, it is suggested that the immune complex deposition in glomeruli can activate the endogenous coagulation system, cause coagulation and fibrinolysis reaction, and eventually promote the pathological damage of kidney.

This study recruited 694 cases of HSPN with a median age of 8.96 years. Types II and III HSPN showed the highest incidence rates, among which IIb and IIIb represented the most common types. There was no significant difference in age distribution among the various pathological HSPN types suggesting renal injury in HSPN progresses rapidly, so it is hard to estimate the extent of injury by the time. Opportune renal biopsy is necessary for renal pathology injury assessment. However, renal biopsy is an invasive operation, which brings trauma and psychological burden to the patients. Therefore, alternative, non-invasive methods/indicators are urgently needed to predict renal pathological types. Here, we showed that 24h urine protein levels and urine protein/creatinine ratios gradually increase with the pathological progression of HSPN. Further analyses showed that urinary protein contents and urine protein/urine creatinine ratios are not significantly different between types I and IIa HSPN patients. Similarly, HSPN type IIb patients had similar urinary protein contents and urine protein/ creatinine ratios compared to types IIIa and IIIb individuals. However, both 24h urinary protein contents and urine protein/creatinine levels were higher in HSPN types IIb, IIIa, and IIIb compared with types I and IIa patients. Similar changes were observed on the levels of eGFR (CKD-EPI) and inflammatory markers among HSPN patients. One of the possible mechanism may be different immune damage degree leads to the different change of glomerular filtration rate among children with HSPN, then influences their urine protein content. These data indicated that 24h urinary protein contents and urine protein/creatinine ratios can be used to predict HSPN type IIb, IIIa and IIIb. Further studies indicate that 24-hour urinary protein level has a higher predictive value than urine protein/creatinine ratio; the areas under ROC curve for these parameters were 0.767 and 0.731, respectively.

In conclusion, cell and humoral immunity, coagulation and fibrinolytic systems are all involved in the pathogenesis of HSPN, and type I hypersensitivity may be the disease trigger of HSPN. 24h-UPRO levels and urine protein/creatinine ratios could probably forecast the pathological classification of HSPN.
